# Experiences of people seen in an acute hospital setting by liaison mental health services: responses from an online survey

**DOI:** 10.1192/bjo.2021.907

**Published:** 2021-06-18

**Authors:** Daniel Romeu, Elspeth Guthrie, Carolyn Czoski-Murray, Samuel Relton, Andrew Walker, Peter Trigwell, Jenny Hewison, Robert West, Mike Crawford, Matt Fossey, Claire Hulme, Allan House

**Affiliations:** 1 Leeds Institute of Health Sciences, University of Leeds, Leeds and York Partnership NHS Foundation Trust; 2 Leeds Institute of Health Sciences, University of Leeds; 3 Clinical Research Network National Coordinating Centre, National Institute of Heath Research; 4 National Inpatient Centre for Psychological Medicine, Leeds and York Partnership NHS Foundation Trust; 5 Department of Brain Sciences, Faculty of Medicine, Imperial College, College Centre for Quality Improvement, Royal College of Psychiatrists; 6 Veterans and Families Institute for Military Research, Faculty of Health, Social Care and Education, Anglia Ruskin University; 7 College of Medicine and Health, University of Exeter

## Abstract

**Aims:**

Recently the NHS has expanded the provision of liaison mental health services (LMHS) to ensure that every acute hospital with an emergency department in England has a liaison psychiatry service. Little work has been undertaken to explore first-hand experiences of these services. The aim of this study was to capture service users’ experiences of LMHS in both emergency departments and acute inpatient wards in the UK, with a view to adapt services to better meet the needs of its users.

**Method:**

This cross-sectional internet survey was initially advertised from May-July 2017 using the social media platform Facebook. Due to a paucity of male respondents, it was re-run from November 2017-February 2018, specifically targeting this demographic group. 184 people responded to the survey, of which 147 were service users and 37 were service users’ accompanying partners, friends or family members. The survey featured a structured questionnaire divided into three categories: the profile of the respondent, perceived professionalism of LMHS, and overall opinion of the service. Space was available for free-text comments in each section. Descriptive analysis of quantitative data was undertaken with R statistical software V.3.2.2. Qualitative data from free-text comments were transcribed and interpreted independently by three researchers using framework analysis; familiarisation with the data was followed by identification of a thematic framework, indexing, charting, mapping and interpretation.

**Result:**

Opinions of the service were mixed but predominantly negative. 31% of service users and 27% of their loved ones found their overall contact with LMHS useful. Features most frequently identified as important were the provision of a 24/7 service, assessment by a variety of healthcare professionals and national standardisation of services. Respondents indicated that the least important feature was the provision of a separate service for older people. They also expressed that a desirable LMHS would include faster assessments following referral from the parent team, clearer communication about next steps and greater knowledge of local services and third sector organisations.

**Conclusion:**

Our survey identified mixed responses, however service users and their loved ones perceived LMHS more frequently as negative than positive. This may be attributed to the recent governmental drive to assess, treat and discharge 95% of all patients seen in emergency departments within four hours of initial attendance. Additionally, dissatisfied service users are more likely to volunteer their opinions. The evaluation and adaptation of LMHS should be prioritised to enhance their inherent therapeutic value and improve engagement with treatment and future psychiatric care.

